# Early childhood traffic-related air pollution and risk of allergic rhinitis at 2–4 years of age modification by family stress and male gender: a case-control study in Shenyang, China

**DOI:** 10.1186/s12199-021-00969-7

**Published:** 2021-04-17

**Authors:** Shuai Hao, Fang Yuan, Pai Pang, Bo Yang, Xuejun Jiang, Aihui Yan

**Affiliations:** Department of Otolaryngology, First Affiliated Hospital of China Medical University, No. 155, Nanjing North Street, Heping District, Shenyang, 110001 China

**Keywords:** Air pollution, Allergic rhinitis, Preschool children

## Abstract

**Background:**

Few studies have explored the modifications by family stress and male gender in the relationship between early exposure to traffic-related air pollution (TRAP) and allergic rhinitis (AR) risk in preschool children.

**Methods:**

We conducted a case-control study of 388 children aged 2–4 years in Shenyang, China. These children AR were diagnosed by clinicians. By using measured concentrations from monitoring stations, we estimated the exposures of particulate matter less than 10 μm in diameter (PM_10_), nitrogen dioxide (NO_2_), ozone (O_3_), carbon monoxide (CO), and sulfur dioxide (SO_2_) in preschool children aged 2–4 years. After adjusted potential confounding factors, we used logistic regression model to evaluate the odds ratio (OR) and 95% confidence interval (CI) for childhood AR with exposure to different air pollutants according to the increasing of the interquartile range (IQR) in the exposure level.

**Results:**

The prevalence of AR in children aged 2–4 years (6.4%) was related to early TRAP exposure. With an IQR (20 μg/m^3^) increase in PM_10_ levels, an adjusted OR was significantly elevated by 1.70 (95% CI, 1.19 to 2.66). Also, with an IQR (18 μg/m^3^) increase in NO_2_, an elevated adjusted OR was 1.85 (95% CI, 1.52 to 3.18). Among children with family stress and boys, PM_10_ and NO_2_ were positively related to AR symptoms. No significant association was found among children without family stress and girls.

**Conclusions:**

Family stress and male gender may increase the risk of AR in preschool children with early exposure to PM_10_ and NO_2_.

## Background

Allergic rhinitis (AR) is the most common chronic allergic disease, involving inflammation of nasal eosinophils caused by air allergen sensitized by IgE [1]. AR is also considered a global health problem. Recently, the prevalence and incidence of AR have been increasing worldwide, especially in developing countries [2, 3]. AR will not cause very serious damage to the body, and preschool children cannot clearly express their condition. Therefore, children with AR are easy to be ignored, especially for some Chinese children [3]. If AR continues to develop, it will cause more serious complications in the upper and lower respiratory tracts, including asthma and sinusitis [4], which will seriously affect the child’s physical and social activities [5]. The etiology of AR is related to genetic and environmental risk factors. Since the rapid increase in the prevalence of AR in children is unlikely to be attributed solely to genetic changes, environmental factors have largely contributed to the development and deterioration of AR in China in the past decade [3]. Environmental factors include physical and social environmental exposure. However, there is little information about the potential effect modification of the social environment (family stress) on the relationship of children AR to the natural environment (traffic-related air pollution).

As a physical environmental factor, traffic-related air pollution (TRAP) can reduce lung function, cause asthma exacerbation, and lead to the development and exacerbation of allergic diseases, such as eczema, asthma, AR, and sensitization [6–8]. A meta-analysis showed that the prevalence of children AR increased with exposure to nitrogen dioxide (NO_2_), sulfur dioxide (SO_2_), and particulate matter (PM_10_ and PM_2.5_), but the relationship between SO_2_/PM_10_ and prevalence of AR was not obviously closely in Asia [9]. Air pollutants come from many emission sources in cities, such as automobile exhaust, household oil fume, and industrial waste gas. In the past 10 years, the air pollution situation in Shenyang has changed. Motor vehicle traffic emissions are the main source of air pollution [10]. TRAP is the main source of changes in the concentration of air pollutants in cities [11]. A cohort study showed TRAP exposure during pregnancy and the first year of life was related to the development of AR in preschool children [3]. In addition, a case-control study showed that for residents less than 75 m from the main road increased lifetime diagnosis and AR symptoms of children aged 6 to 14 [12]. However, previous studies showed no significant correlation between TRAP exposure in early years of life and AR prevalence in children aged 1–12 years [13, 14]. These inconsistencies in the epidemiological survey results are mainly due to differences in TRAP exposure assessment and the classification of the subgroups included. These studies assessed TRAP exposure through traffic measurements or modeling air pollutant concentrations, and most studies only considered the home address at birth [3, 12–14]. Furthermore, these studies included children aged 1–12 years and rarely included a narrow subgroup. Therefore, we need to limit the air exposure window of children with AR and the key components of air pollution in order to develop more effective prevention and intervention measures.

As a social environmental factor, psychological stress is related to allergic diseases including rhinitis, rhinoconjunctivitis, allergic conjunctivitis, wheezing, and asthma in children [15–18]. In recent years, little consideration has been given to the psychological impact of family stress on preschool children in China [19]. This is mainly because under the control of traditional Chinese culture, preschool children are unable or unwilling to express their feelings to their parents. Especially, in the face of stress, the depressive performance of parents will damage the entire family environment, thereby inhibiting the psychosocial functions of preschool children [20]. Family stress (including separation/divorce of parents, unemployment of parents, serious health problems, death of family members or close relatives) is a risk factor closely related to the occurrence or development of asthma [21, 22]. This may be due to being stimulated by antigen or stimulating immune cells in vitro [21, 23, 24]. However, few studies have explored the moderating effects of family stress and gender on the occurrence of AR in preschool children under TRAP. Therefore, this study aimed to evaluate whether preschool children are prone to develop AR during early-life TRAP exposure including PM_10_, NO_2_, ozone (O_3_), carbon monoxide (CO), and SO_2_ exposures, with emphasis on stress or other possible influencing factors.

## Methods

### Study design and participants

We conducted a case-control study on TRAP-induced AR among preschool children aged 2–4 years in Shenyang, the capital of Liaoning Province in Northeast China. Liaoning province has a population of 8.32 million and an area of 12,948 km^2^. All protocols were approved by the medical ethics committee of China Medical University [approval number 2013-01-013]. Since 2013, we have carried out a long-term study of Children’s respiratory health (CRH) screening in Shenyang. These main data come from the cohort study (CRH) for pregnant women at First Affiliated Hospital of China Medical University, from April 2013 to September 2014. The birth cohort included single births, full-term newborns, without deformities, and excluded infants whose mothers were younger than 20 years old and had alcohol or drug addiction. The data for this study came from Department of Obstetrics of the First Affiliated Hospital. We collected information about interviews with the mothers of each enrollee and telephone interviews with their parents when the children were 2, 3, and 4 years old. If the questionnaire showed AR symptoms, we invited these children to hospital for further diagnosis and free treatment. We determined the incidence of AR in preschool children in the birth cohort, and then their parents filled out the questionnaire when the children were 2, 3, and 4 years old. Every year, the professional doctors of our research group visited the parents of the included children by telephone to assess whether their children had AR in the one year before the diagnosis, and we recorded the data of TRAP exposure before the diagnosis of AR. We did not collect data that included children aged 1 year, because Chinese parents are reluctant to follow up their children by phone before their first birthday unless they have very obvious symptoms. In addition, the parents rarely paid attention to AR symptoms from birth to 2 years old, so we did not collect complete data. Thus, in this study, we selected children aged 0–2 years without AR symptoms, and calculated the average exposure level from 2 years of age to the day of AR diagnosis.

Here, the enrolled preschool children have always lived in their birthplace. From 2017 to 2018, we invited 5706 children aged 2–4 years and their mothers to participate in AR screening. These parents of enrolled children signed an information and consent form. The parents of the children participating in this study have read and signed the information and consent form before screening. Since the concentration of TRAP may decrease as the increase of building height, we excluded those children who lived above the fourth floor (about 8 m from the ground) [14]. In addition, in traditional Chinese families, most children go to kindergarten from the age of three. The resulting change in location (home and kindergarten) may increase personal contact errors. Therefore, we excluded those children whose homes were more than 400 m away from the kindergarten. In fact, we intended to make the air pollution of the children’s home and kindergarten as same as possible. Since Chinese parents usually choose the kindergarten closest to their home [3], this situation can reduce the misclassification of personal contact.

In fact, we invited 5706 families (100%) to participate in the survey, 1211 (21.2%) families did not fill out the questionnaire, and 36 (0.6%) families had hidden information. Then, 4459 families (78.2%) completed the questionnaire and received the diagnosis. After that, a total of 3047 children’s parents completed a symptom questionnaire about demographic information and daily life habits and received a diagnosis from a clinician. Moreover, these parents provided their children’s information on the clinical symptoms of AR in the previous year (“In the past 12 months, did your child have a sneezing problem, or a runny or stuffy nose without a cold or flu?”). In addition, these parents also provided information about children with clinical symptoms of AR in the previous year (“In the past 1 year, did your children have a sneezing problem, or a runny nose or nasal congestion without a cold or flu?”). Other current socioeconomic factors were provided through the use of the Chinese revised version of “AR International Research in the children’s standardized questionnaire”.

In the end, we recruited 194 participants with AR (case group) and 194 participants without AR (control group) from 3047 children. The case group needs to be diagnosed by otolaryngologists and medical staff in accordance with the “Chinese AR Allergen Immunotherapy Guidelines”, excluding children with asthma, atopic dermatitis, or allergic conjunctivitis [1]. The control group was randomly selected from other children (*n* = 2853), without allergic diseases (including allergic conjunctivitis, AR, asthma, and atopic dermatitis), and matched with cases (1:1) by month. Air pollution exposure of the individual participants (*n* = 388) was estimated based on the measurement results of the municipal air monitoring stations, which was located about 1000 m from each participant’s home, and these monitoring points in Shenyang are mainly located in major traffic near the roads. The relative distance between the participant’s address and the location of the emission source affect the levels of air pollutants. We recorded the addresses of each child’s home and kindergarten, and then selected the nearest monitoring station from the line between the family and the kindergarten to assess traffic air pollution exposure. If the relative distance exceeded 1000 m, the average of five nearby monitoring stations was used as the representative value. In order to reduce the bias caused by the distance between the family and the kindergarten, we selected children whose family is within 400 m from the kindergarten. In addition, since this study only analyzed the existing data of indirect interaction with the subjects, this study complies with the principle of giving up informed consent.

### TRAP exposure

According to the related epidemiological studies on TRAP and allergic diseases [3, 5], we selected five pollutants in Shenyang, namely PM_10_ (beta gauge method), SO_2_ (ultraviolet fluorescence method), CO (nondispersive infra-red absorption method), NO_2_ (chemiluminescence method), and O_3_ (ultraviolet absorption method) as TRAP markers [25]. According to the principle that errors will occur if the established statistical model contains highly correlated pollutants, and the pilot test also reflected that there was a high correlation between PM_2.5_ and PM_10_ (*r* = 0. 841), and PM_2.5_ are sub-elements of PM_10_. So PM_2.5_ was not chosen in this study. Based on the previous study [26], we calculated the average ambient concentrations of PM_10_, O_3_, SO_2_, CO, and NO_2_. We obtained the average daily concentrations of these pollutants for each participant from 2015 to 2018, selected children between 0 (2013/2014) and 2 years old (2015/2016) without AR symptoms, and calculated the average daily concentrations of these pollutants for each child from 2 years old to the day of AR diagnosis. We recorded TRAP exposure from April 2015 to September 2018. The data came from air pollution monitoring station within 1000 m of each participant’s residence. We recorded the daily average exposure of each child in usual residence and then calculated the monthly average concentration of the aggregate exposures. As for the exposure of CO and O_3_, we calculated the average 8-h concentration from these same monitors.

### Covariates and stress measure

Through a survey of parents of enrolled children, we collected the characteristics of children (sex, birth weight, eczema, and feeding mode in the first year), the characteristics of parents (delivery mode, parents’ allergy history: at least one parent has a history of AR, active and passive smoking), socioeconomic status of the family (address, pets, furniture, redecoration, and family stress including parental separation/divorce, parental unemployment, serious health problems, or the death of a family member or close relative). Children’s chronic stress was obtained through the Life Stress Interview from UCLA [27].

### Statistical analysis

We used Chi-square test to compare the frequencies in the baseline characteristics of two groups, used two-sample *t*-test to compare the birth weight values of the two groups, and used Logistic regression model to evaluate the covariates increasing the correlation of TRAP and AR risk. If the covariate is significantly correlated with risk (*p* < 0.05), or the adjusted odds ratio (aOR) changes by more than 10%, then the covariate is selected to enter the model analysis. To evaluate the existence of potential moderating effects, we used likelihood ratio test (LRT) to compare models with and without moderating variables in the conditional logistic regression model. If there is a significant level of LRT difference (*p* < 0.1), it is considered that there is a moderating effect. Only family stress and male gender met this criterion, so only the stratified OR of these two moderating variables was shown. We used aOR and 95% confidence interval (95% CI) to express the relationship between TRAP and AR. The recorded TRAP levels were continuous variables. We used quartiles to represent the relationship between the increase in PM_10_, SO_2_, NO_2_, CO, or O_3_ and AR. We chose exposure levels below the 25th percentile as the reference (baseline level) for each pollutant. Then, through the interquartile range (IQR), the continuous variables were changed to the categorical variables, and the linear correlation between the pollutant parameters was analyzed for the co-linearity test. The data were analyzed by SPSS software for Windows, version 20.0 (IBM SPSS, Inc., Chicago, IL, USA).

## Results

### Participants

**Fig. 1:**
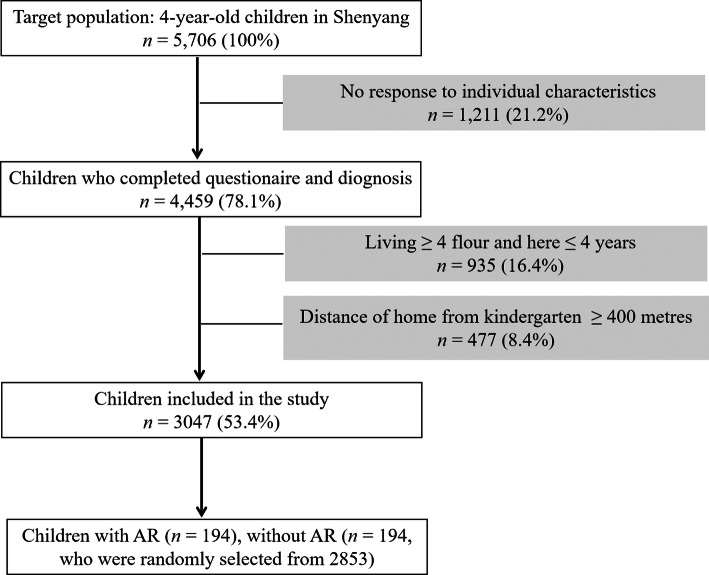
The flow chart of cases and controls selection

Table 1 showed the natural characteristics and clinical diagnosis of 3047 children. Taking into account the living floor, the distance between the family and the kindergarten, we deleted 40% of the children (*n* = 2,659). Compared with the 2659 children who were not included in the study, the parents of 3047 children who were included were more likely to have a history of AR (*p* = 0.01). There was no statistical differences in other demographic variables between the children who were not included and the children who were included in the study. Figure 1 was a flowchart of cases and controls selection.

**Table 1:** Baseline characteristics of children included (*n* =3047) and not included (*n* = 2659)

Baseline characteristics	Included *n* (%)	Not included *n* (%)	*p*-value
Male sex	1569 (51.5)	1377 (51.8)	0.79
Birth weight	3.45 ± 0.43	3.45 ± 0.47	0.83
Residence in city	2163 (71.0)	1808 (68.0)	0.46
Environmental smoke	768 (25.2)	723 (27.2)	0.61
Cesarean	2142 (70.3)	1840 (70.4)	0.95
Formula-feeding	985 (32.6)	863 (33.1)	0.64
Parental allergy	703 (23.1)	361 (13.8)	0.01
Pets	693 (23.0)	606 (23.2)	0.92
House redecoration	250 (8.4)	195 (7.7)	0.75
Stressful family	605 (20.3)	541 (21.1)	0.68

Total numbers may not be equal to 3047 and 2659 for some characteristics due to missing data

### PM_10_, SO_2_, NO_2_, CO, and O_3_ levels

Table 2 showed the pollutants (PM_10_, SO_2_, NO_2_, CO, and O_3_) concentration distribution in the included children before AR diagnosis date. The concentration ranges of these pollutants (PM_10_, SO_2_, NO_2_, CO, and O_3_) were from 40 to 115 μg/m^3^ with a median (IQR) of 88 (75–95) μg/m^3^, from 5 to 59 μg/m^3^ with a median of 26 (20–34) μg/m^3^, from 12 to 60 μg/m^3^ with a median of 31 (23–41) μg/m^3^, from 480 to 2380 μg/m^3^ with a median of 970 (906–1260) μg/m^3^, and from 63 to 116 μg/m^3^ with a median of 92 (80–102) μg/m^3^, respectively.

**Table 2:** Distribution of ambient air pollution concentrations (μg/m^3^)

Air pollutant	Mean	Percentile distribution			IQR
		*P*_25_	*P*_50_	*P*_75_	
PM_10_	88	75	88	95	20
SO_2_	26	20	26	34	14
NO_2_	31	23	31	41	18
CO	970	906	970	1260	354
O_3_	92	80	92	102	22

*IQR* interquartile range

### Main results

Table 3 showed the distribution of sociodemographic characteristics of AR children compared to controls. The 6.4% (194/3,047) among enrolled children aged 2–4 years was diagnosed by clinicians. There were statistical differences between cases and controls in the distributions of the following characteristics: percentage of boys (64% vs 48%), city residence (83% vs 70%), eczema in the first year after birth (57% vs 41%), parental allergy (31% vs 13%), and family stress (50% vs 18%). There was no statistically significant difference in the distribution of other variables between cases and controls. Overall, the crude ORs for AR was 2.18 (95% CI, 1.35 to 3.52) for children living in cities, 1.88 (95% CI, 1.26 to 2.81) for boys, 1.91 (95% CI, 1.28 to 2.85) for children suffering from eczema in the first year after birth, 2.94 (95% CI, 1.77 to 4.87) for children with a history of parental allergies, and 4.14 (95% CI, 2.65 to 6.49) for children with family stress. There were no significant associations of AR with other variables, no co-linearity problems for TRAP and others parameters.

**Table 3:** Characteristics of children with or without allergic rhinitis (*n* = 388)

Characteristics^*a*^	Cases (*n* = 194), *n* (%)	Controls (*n* = 194), *n* (%)	Crude odds ratio (95% CI)	*χ*^2^	*p*-value
Sex					
Girls	70 (36.1)	101 (52.1)	Reference	9.557	0.002
Boys	124 (63.9)	93 (47.9)	1.880 (1.258, 2.809)		
Birth weight	3.53 ± 0.59	3.40 ± 0.67	-	-	0.844
Residence					
Suburbs	33 (17.0)	59 (30.4)	Reference	10.455	0.001
Inner city	161 (83.0)	135 (69.6)	2.180 (1.352, 3.516 )		
Environmental tobacco smoke at home					
No	142 (73.2)	151 (77.8)	Reference	0.567	0.452
Yes	52 (26.8)	43 (22.2)	1.190 (0.756, 1.873)		
Mode of delivery					
Vaginal delivery	58 (29.9)	55 (28.4)	Reference	0.011	0.916
Elective cesarean section	136 (70.1)	139 (71.6)	0.977 (0.635, 1.502)		
Mode of feeding in the first year					
Breast-feeding	129 (66.5)	133 (68.6)	Reference	0.060	0.807
Formula-feeding	65 (33.5)	61 (31.4)	1.054 (0.693, 1.601)		
Eczema during the first year					
No	83(42.8)	115 (59.3)	Reference	10.238	0.001
Yes	111(57.2)	79 (40.7)	1.910 (1.283, 2.845)		
Parental history of allergy					
No	134 (69.1)	171 (88.1)	Reference	18.223	0.000
Yes	60 (30.9)	23 (11.9)	2.935 (1.769, 4.872)		
Furry/feathery pets					
No	150 (77.3)	151 (77.8)	Reference	0.040	0.841
Yes	44 (22.7)	43 (22.2)	0.953 (0.598, 1.520)		
New furniture after birth					
No	98 (50.5)	103 (53.1)	Reference	0.234	0.628
Yes	96 (49.5)	91 (46.9)	1.102 (0.744, 1.633)		
House redecoration during the past year					
No	171 (88.1)	180 (92.8)	Reference	2.334	0.127
Yes	23 (11.9)	14 (7.2)	1.695 (0.856, 3.354)		
Stressful family events during the first 2 years^*b*^					
No	98 (50.5)	160 (82.5)	Reference	40.910	0.000
Yes	96 (49.5)	34 (17.5)	4.144 (2.647, 6.489)		

Data are shown as *n* (%) or mean ± SD

^*a*^Characteristics are at birth unless otherwise specified

^*b*^Among parental separation/divorce, parental loss of job, serious health problem, or death of a family member or close relative

Table 4 showed the Spearman correlation coefficients of these pollutants (PM_10_, SO_2_, NO_2_, CO, and O_3_) concentrations before the diagnosis of AR. These values indicated that there was a moderate correlation between the average values of these pollutants.

**Table 4:** Spearman correlation coefficients for the estimates of ambient air pollutant concentrations

Air pollutant	PM_10_	SO_2_	NO_2_	CO	O_3_
PM_10_	1	0.442	0.463	0.413	0.165
SO_2_		1	0.339	0.452	− 0.282
NO_2_			1	0.709	− 0.210
CO				1	− 0.235
O_3_					1

Table 5 showed the estimated impact of pollutants (PM_10_, SO_2_, NO_2_, CO and O_3_) on the occurrence of AR in preschool children with each additional IQR under single factor analysis. In the single-pollutant models, under the premise of adjusting for other influencing factors, AR was significantly correlated with PM_10_ and NO_2_ in preschool children, with aOR = 1.31 (95% CI, 1.08 to 1.90) and 1.15 (95% CI, 1.02 to 2.23) respectively. Regardless of the single-pollutant models or the multi-pollutant models, AR was statistically related to PM_10_ and NO_2_. In the multi-pollutant models, the correlations between these pollutants (PM_10_, NO_2_, O_3_, and CO) and AR were enhanced, accompanied by a wider CI range. Only the OR attenuation between SO_2_ and AR was close to the null value. Thus, the correlation between the pollutant (PM_10_ or NO_2_) and AR was strong in preschool children, apart from affecting the changes in the effect estimation.

**Table 5:** Conditional logistic regression estimated adjusted odds ratios (ORs) and 95% confidence intervals (CIs) for associations of allergic rhinitis with ambient air pollution exposure among children

	Single^*a*^	Multi^*b*^
PM10	1.31 (1.08, 1.90)*	1.70 (1.19, 2.66)*
SO2	1.26 (0.73, 1.97)	0.84 (0.49, 1.38)
NO2	1.15 (1.02, 2.23)*	1.85 (1.52, 3.18)*
CO	1.13 (0.77, 2.02)	1.15 (0.64, 2.39)
O3	0.52 (0.23, 1.02)	0.60 (0.31, 1.08)

OR (95% CI) was estimated for an IQR increase in PM10, SO2, NO2, CO, and O3

*p < 0.05

**p < 0.01

^*a*^Single-pollutant model: adjustment for all the potential covariates in Table 2 including personal factors (sex, birth weight, delivery, feeding, eczema, parental allergy, and stress) and indoor factors (tobacco smoke, new furniture, pets, house redecoration)

^*b*^Multi-pollutant model: PM_10_ + SO_2_ + NO_2_ + CO + O_3_. Further adjustment for the effects of the other air pollutants on the base of single-pollutant model

Table 6 showed the logistic regression of the relationship between AR and pollutants (an IQR increase in PM_10_ and NO_2_) was stratified by gender, eczema, heredity, or stress after other related variables were adjusted. The bases for stratification were based on the variables in Table 3 that had statistically different associations with AR. An IQR (20 μg/m^3^) increase in PM_10_ in boys was positively statistically associated with the risk of AR with aOR = 1.46 (95% CI, 1.13 to 2.08), and 1.76 (95% CI, 1.10 to 3.11) among preschool children with stress, respectively. Moreover, an IQR (18 μg/m^3^) increase in NO_2_ in boys was positively statistically associated with the risk of AR with aOR = 2.13 (95% CI, 1.31 to 3.45), and 1.94 (95% CI, 1.18 to 3.20) among preschool children with stress.

**Table 6:** Association (adjusted odds ratios and 95% confidence interval) between exposure and allergic rhinitis among children (*n* = 388), stratified by significant covariates

	*n*	Effect per 20 μg/m^3^ of PM_10_	Effect per 18 μg/m^3^ of NO_2_
		aOR (95% CI), *p*-value^a^	aOR (95% CI), *p*-value^a^
Stressful family events			
No	258	0.93 (0.63, 1.22)	0.94 (0.73, 1.84)
Yes	130	1.76 (1.10, 3.11)*, 0.028	1.94 (1.18, 3.20)**, 0.009
Parental history of allergy			
No	205	0.80 (0.61, 1.08)	1.09 (0.63, 1.23)
Yes	83	1.08 (0.95, 1.34), 0.113	1.46 (1.00, 2.26), 0.084
Eczema during the first year			
No	198	0.76 (0.49, 1.20)	0.91(0.28, 1.85)
Yes	190	0.91 (0.55, 1.52), 0.121	1.05 (0.69, 1.60), 0.098
Gender			
Girls	171	0.95 (0.53, 1.36)	1.42 (0.92, 2.10)
Boys	217	1.46 (1.13, 2.08)*, 0.039	2.13 (1.31, 3.45)**, 0.004

OR (95% CI) was estimated for an IQR increase in PM_10_, SO_2_, NO_2_, CO, and O_3_

*p < 0.05

**p < 0.01

Adjustment for all the potential covariates in Table 2 including personal factors (sex, birth weight, delivery, feeding, eczema, parental allergy, and stress) and indoor factors (tobacco smoke, new furniture, pets, house redecoration) except for the stratification variables

^a^*p*-value of likelihood ratio test comparing model fit with and without inclusion of interaction terms

Table 7 showed that family stress and male gender were two moderating variables that positively correlated pollutant exposure (PM_10_, NO_2_) with the risk of AR. In the single-pollutant model, these factors interacted on a multiplicative scale. Stratified by family pressure or gender, there was no dose-response relationship of the positive correlation between PM_10_ and the risk of AR. However, there was a dose-response relationship between NO_2_ and AR. In the single-pollutant models, family stress increased the positive correlation between pollutants (PM_10_, NO_2_) and the risk of AR, with 1.80 (95% CI, 1.61 to 2.02) and 1.98 (95% CI, 1.74 to 2.43) for the above 75th percentile vs. below the 25th percentile of exposure, than among never family stress [1.07 (95% CI, 0.86 to 1.36), 1.48 (95% CI, 1.03 to 1.85)], respectively. According to the effect evaluation of LRT heterogeneity, there was statistical significance (*p* = 0.03, 0.04). Also, boys increased the positive correlation between pollutants (PM_10_, NO_2_) and AR, with 1.49 (95% CI, 1.18 to 1.82) and 2.10 (95% CI, 1.85 to 2.55) for the above 75th percentile vs. below the 25th percentile of exposure, than among girls [1.11 (95% CI, 0.93 to 1.42) and 1.52 (95% CI, 1.21 to 1.94)], respectively ( *p* = 0.01, 0.01).

**Table 7:** Conditional logistic regression estimated adjusted odds ratios (aORs) and 95% confidence intervals (CIs) for associations of allergic rhinitis with air pollutant concentrations, stratified by stress and gender

Pollutant	Quintile	Stressful family events	Gender
		No, yes	Girls, boys
		aOR (95% CI), aOR (95% CI) *p*-value^a^	aOR (95% CI), aOR (95% CI) *p*-value^a^
PM_10_	1	Reference, reference	Reference, reference
	2	0.94 (0.81, 1.13), 1.70 (1.54, 1.92)	1.02 (0.83, 1.30), 1.41 (1.01, 1.77)
	3	0.91 (0.71, 1.28), 1.63 (1.41, 1.87)	0.95 (0.74, 1.23), 1.38 (1.00, 1.65)
	4	1.07 (0.86, 1.36), 1.80 (1.61, 2.02) 0.03	1.11 (0.93, 1.42), 1.49 (1.18, 1.82) 0.01
NO_2_	1	Reference, reference	Reference, reference
	2	1.05 (0.84, 1.31) 1.89 (1.52, 2.13)	1.23 (1.04, 1.58), 1.51 (1.18, 1.95)
	3	1.16 (0.93, 1.57) 1.90 (1.47, 2.25)	1.36 (1.17, 1.66), 1.78 (1.26, 2.20)
	4	1.48 (1.03, 1.85) 1.98 (1.74, 2.43) 0.04	1.52 (1.21, 1.94), 2.10 (1.85, 2.55) 0.01

1 = below the 25th percentile, 2 = between the 25th and 50th percentiles, 3 = between the 50th and 75th percentiles, 4 = above the 75th percentile. Adjustment for all the potential covariates in Table 2 including personal factors (sex, birth weight, delivery, feeding, eczema, parental allergy, and stress) and indoor factors (tobacco smoke, new furniture, pets, house redecoration) except for the stratification variables

^a^*p*-value of likelihood ratio test comparing model fit with and without inclusion of interaction terms

## Discussion

In this case-control study, we explored the moderating effects of family stress on the relationship between early-life TRAP exposure and the risk of AR in preschool children. In the whole study population, PM_10_ or NO_2_ exposure was positively associated with the risk of AR in children aged 2–4 years, respectively. We also analyzed whether sex, parental allergy history, and eczema in the first year increased the risk of AR in preschool children, under PM_10_ or NO_2_ exposure. These factors were also positively correlated with AR. By exploring the potential moderating effects of numerous risk factors associated with AR (when a causal effect of intervening on one exposure, there was another variable stratification), we found that family stress or boys increased the risk of PM_10_ or NO_2_ exposure to AR.

The main findings of this study were to explore the relationship between early TRAP (PM_10_ or NO_2_) exposure and the risk of AR in preschool children. Family stress and male gender may play a moderating effect on the relationship. Using the multi-pollutant model, the risks of other air pollutants to AR were adjusted to estimate their correlation. PM_10_, SO_2_, NO_2_, CO, and O_3_ have been used as markers of traffic air pollution. We only found that preschool children exposed to PM_10_ and NO_2_ had an increased risk of AR by 70% and 85%, respectively.

The prevalence of AR among middle-school students in Taiwan was related to the concentrations of nitrogen oxides (NOx) and CO exposures [25]. In China, atmospheric CO and NO_2_ are the emissions of automobile exhaust, which are caused by incomplete combustion inside motor engines [11]. We used a multi-pollutant model to evaluate the association between combined exposure (PM_10_, O_3_, SO_2_, CO and NO_2_) and the risk of AR in children, instead of choosing PM_2.5_ as a marker of TRAP. Because PM_2.5_ is highly correlated with PM_10_, there may be some potential variance inflation and deviation [28, 29]. Since we did not know which model would cause the least bias in the estimation, we compared the results of the single-pollutant model and the multi-pollutant model. In most cases, in both models, the effect estimates show similar associations between exposure and dependent variables. And most of the effect estimates in the multi-pollutant model are higher than those in the single-pollutant model. This study showed the same performance. The reason was that the multi-pollutant model produced synergistic or additive effects, which was consistent with other reports on air pollution and childhood allergic diseases [3, 30]. Therefore, in future research, we need to accurately evaluate the effect and improve the evaluation of the concentrations of pollutants and covariates. The complex sources of exposure may cause PM_2.5_ and PM_10_ to be non-specific pollutants and reduce the correlation. But, this study showed that PM_2.5_ and PM_10_ are highly correlated. This aspect may be related to the current main source of air pollution in China is automobile exhaust [10]. On the other hand, it may be because PM can act as an allergen carrier, exacerbating allergic reactions, and smaller particles (PM_2.5_) can enter the respiratory tract of underdeveloped lungs and anatomically smaller peripheral airways more quickly in children [9, 31, 32]. Moreover, in China, heavy metal elements (S, Zn, Cu, and Pb) account for a large proportion of PM_10_, while S and Pb are relatively enriched in PM_2.5_ [33]. The difference in chemical compositions of PM may cause different degrees of mucosal damage [9].

The levels of O_3_ and NO_2_ are coupled by chemical bonds. The formation of O_3_ requires sunlight to consume nitrogen [34]. Thus, especially in areas with heavy traffic, when NO_2_ exposure is high, O_3_ exposure is usually low [35–37]. And, NO_2_ can directly cause inflammation in children’s sensitive airways, such as AR [38]. The association between SO_2_ exposure and the risk of AR in children is controversial [9]. From the point of view of the decrease of SO_2_ content or the mixing of multiple pollutants, due to the increase of concentrations in other air pollutants, we cannot fully explain the inconsistent ORs trend of SO_2_ exposure between the single-pollutant model and the multi-pollutant model. Other pollutants may enhance the corrosiveness of SO_2_ [39]. In Asia, most previous studies did not show a statistically significant correlation between SO_2_ concentration and AR prevalence in children. On the contrary, some recent studies showed that SO_2_ concentration was closely related to the prevalence of AR. The change of this trend may be related to different regions, races, and nationalities [9, 39], whereas this debate on the correlation between SO_2_ and the risk of AR in children may be mainly due to allergen-related or neurogenic complex mechanisms [40, 41]. The difference between SO_2_ single exposure and mixed exposure was due to the fine dynamic balance between pro- and anti-apoptotic genes in damaged airway epithelium and undamaged airway epithelium [40]. Unfortunately, we did not detect the related immune indicators including these anti-inflammatory cytokines. Other studies have shown that SO_2_ exposure in multi-pollutant model (petrochemical exposure) had no or moderate association with children’s respiratory diseases [42–44]. Thus, we need to expand the sample size in analyzing the correlation between SO_2_ and children AR.

Because as the height of the building increases, TRAP exposure will decrease [14]. We chose children living below the 4th floor in order to more accurately estimate the actual situation of TRAP exposure. At the same time, to adjust the misclassification bias from TRAP exposure, we collected every child who did not change the family residence during the observation period, and his/her home was very close to the kindergarten, or did not even go to kindergarten. In addition, we adjusted the statistical model for other sources of children’s exposure to TRAP (such as smoking at home).

Respiratory and immune system develop and mature rapidly in early childhood, so TRAP exposure during this sensitive period is more likely to damage children’s health [40, 41]. Thus, we chose the early exposure as the observation window for TRAP exposure to help assess the associations similar to other study, which have shown that in the third trimester and the first year of life, TRAP was more dangerous for asthma, AR, and eczema than exposure later [42]. Moreover, TRAP exposure during early developmental period could trigger AR later (aged 4 years old) [43]. The correlation between TRAP and AR was more obvious in children aged 3–4 years old [3]. Allergic children exposed to TRAP are more likely to develop asthma and allergy than non-allergic children [7, 43]. However, we only found that stress and gender factors may exacerbate the risk of TRAP on AR and did not find the influence of genetic susceptibility on AR. This may be related to the multi-collinearity from the two variables (parental allergy history and infant eczema) in this study. A meta-analysis reports that psychological stress will cause pregnant women to continuously secrete cortisol, which in turn increases the risk of asthma and allergy in offspring [44]. This may be due to the differentiation of dominant T helper 2 (Th2)-biased cell, which causes Th1/Th2 homeostasis imbalance in the immune system [45, 46].Thus, stress may increase the risk of TRAP by disrupting the immune response [47–49], even at lower doses of physical exposure to promote adverse consequences [50, 51]. Whether there is a difference between gender in the associations between TRAP exposure and respiratory diseases is still controversial. For instance, a French birth cohort study showed that there was a positive association between nitrogen oxides and persistent wheezing in 4-year-old boys [46], while a Swedish study showed similar performance in 4-year-old girls [52]. In addition, a meta-analysis of five birth cohorts showed that PM_2.5_ exposure was positively correlated with asthma in boys aged 4–5 years [45]. A Chinese similar cohort study showed a positive correlation between TRAP exposure and AR in boys aged 3–6 years [53]. The above gender differences are due to the later appearance of male lung surfactants in newborns. From the prenatal period to the first year after birth, the respiratory tract of boys is narrower than that of girls [54, 55].

There are several limitations as follows: First, the small sample size may lead to insufficient power to assess an interaction [56]. In order to reduce the possibility of attributable to chance, the investigation needs to be repeated. Second, although we chose the straight-line distance between family and kindergarten to be less than 400 m, there may still be potential exposure misclassifications. Three, the number of monitoring points was scarce and the land use conditions were ignored; thus, the exposure measurement at fixed monitoring points may lead to exposure bias. Four, although in modern Chinese cities with similar scales and architectural styles, the results of this study could be extrapolated to assess the relationship between air pollution and childhood allergic diseases. But, as a northern city in China, the climate and humanistic characteristics of Shenyang are different from those of southern China, so further research is needed to infer these results. Five, this study only involved outdoor air pollution exposure. But because children spend 90% of their time indoors [57] and the concentration of PM_10_ and NO_2_ varies greatly between indoor and outdoor [58, 59], it may cause uncertainty in air pollution exposure [57, 58]. However, at present, urban residents in Shenyang basically use natural gas for cooking, which can reduce indoor smoke and dust pollution; moreover, Chinese families are paying more and more attention to air quality and increasing the time to open windows for ventilation, which also increases outdoor air pollution exposure [10]. It cannot be ignored that Shenyang is a heavily polluted city in the northern of China; we speculate that the serious outdoor air pollution may cover up indoor air pollution [60]. Further work is required to investigate the potential interaction between outdoor or indoor air pollution and AR. Finally, we did not collect full data at 0–1 year of age and only calculated the 2 years of age to the day of AR diagnosis. This made our study inadequate in the ability to identify the susceptible window of exposure in the development of childhood AR and possible effect modifiers. Thus, the next study should be to investigate the relation of early TARP during pregnancy and the first year of life with the development of AR in later life, and possible effect modifiers.

## Conclusion

Our case-control study supports that family stress and male gender may increase the susceptibility of AR caused by early exposure to TRAP (PM_10_ or NO_2_) in preschool children aged 2–4 years.

## Data Availability

The data can be made available from the corresponding author for all interested researchers upon requests sent to the author’s office. The initial contact for request should be addressed to the corresponding author’s institution.

## Abbreviations

AR: Allergic rhinitis
CO: Carbon monoxide
CI: Confidence interval
IQR: Interquartile range
NO_2_: Nitrogen dioxide
OR: Odds ratio
O3: Ozone
PM_10_: Particulate matter less than 10 μm in diameter
SO_2_: Sulfur dioxide
TRAP: Traffic-related air pollution
